# Supplementary material for: Hydrogel Films Based on Chitosan and Oxidized Carboxymethylcellulose Optimized for the Controlled Release of Curcumin with Applications in Treating Dermatological Conditions
Source: Molecules. 2021 Apr 10;26(8):2185. doi: 10.3390/molecules26082185 (PMC8069243; doi:10.3390/molecules26082185)
Supplement: Supplementary file 1 [file molecules-26-02185-s001.pdf]

Supplementary materials

Manuscript Title: "Hydrogel films based on chitosan and oxidized carboxymethylcellulose optimized for the controlled release of curcumin with applications in treating dermatological conditions"

Authors: Mohammed DELLALI, Camelia Elena IURCIUC (TINCU), Corina Lenuța SAVIN, Nawel SPAHIS, M'hamed DJENNAD, Marcel POPA

Figure S1:

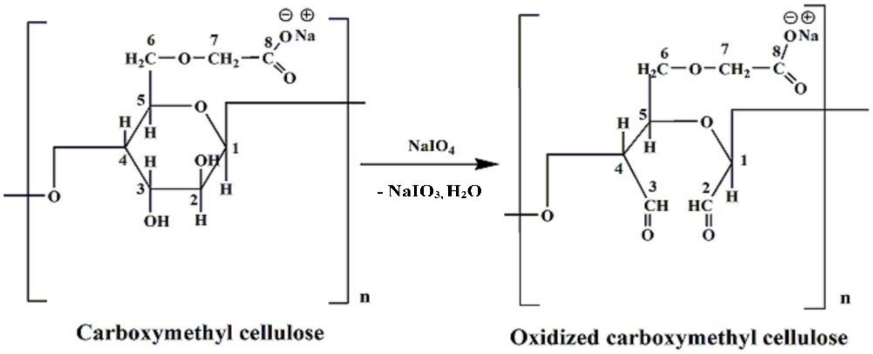

Figure S1: Schematic representation of CMC's oxidation reaction under the sodium periodate action.

Figure S2:

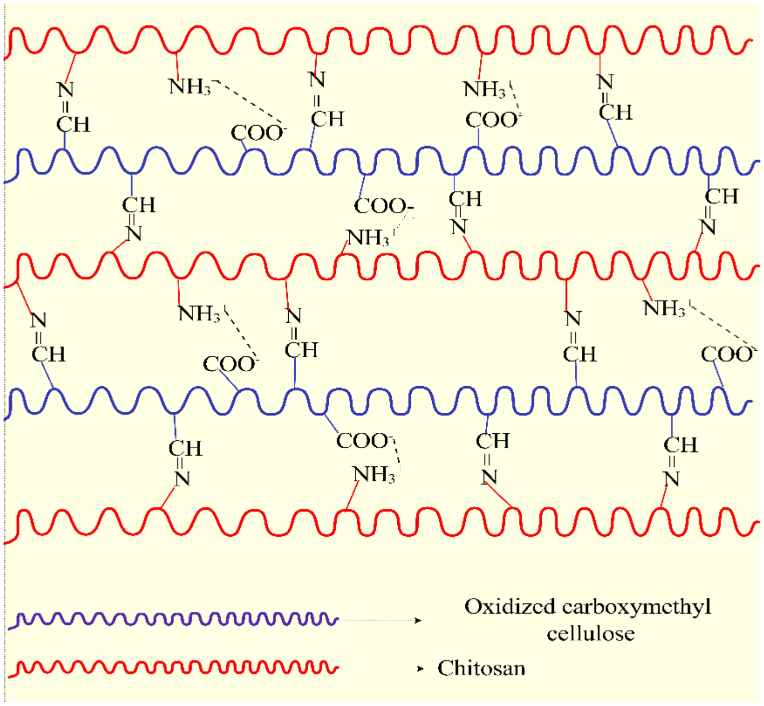

Figure S2. The structure of the hydrogel films based on chitosan and oxidized carboxymethyl cellulose - schematic presentation

Figure S3

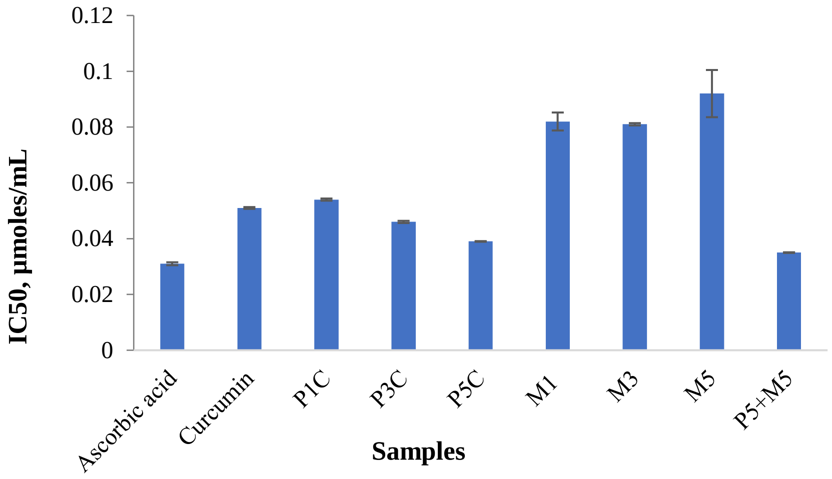

Figure S3. Antioxidant activity determination expressed by IC50 values for the analyzed samples using the DPPH assay.

Figure S4:

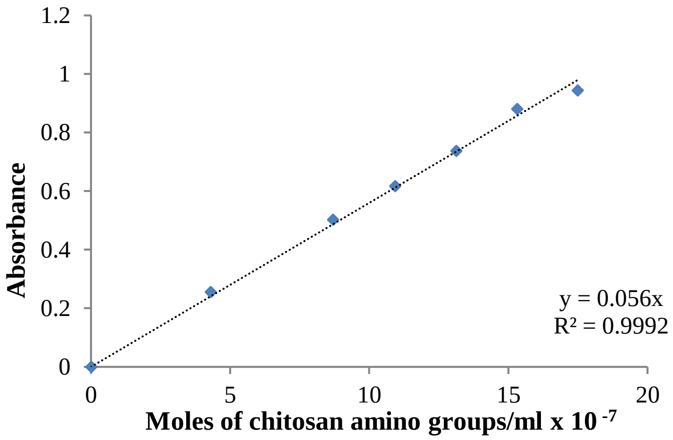

Figure S4: The CS calibration curve determined with ninhydrine test

Figure S5

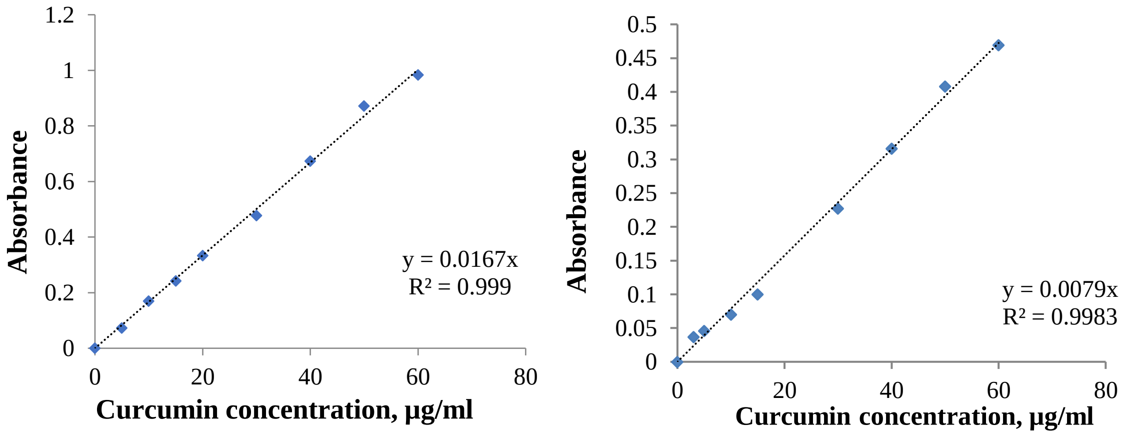

(a) (b)

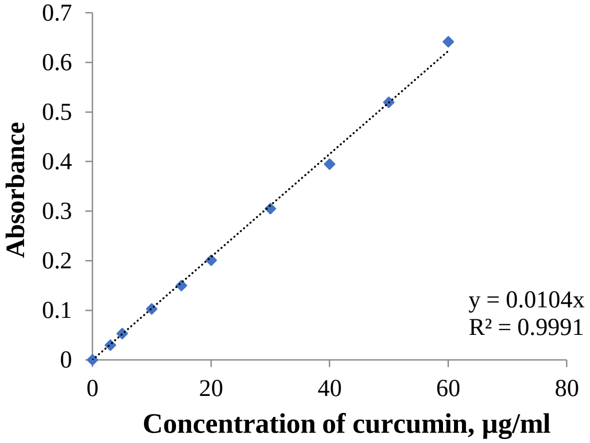

(c)

Figure S5. Calibration curves of curcumin in ethanol (a), phosphate buffer at pH=7.4 (b), and acetate buffer at pH=5.5 (c)

Table S1.

Table SI. The CI values (%) for samples obtained by chemical cross-linking and physical interaction between CS and CMCOx, respectively, by the CS amino groups' interaction with CMC's carboxylic groups.

Samples	Molar ratios	$CI_{\text{chemical cross-linking and physical interactions}}$ (%)	$CI_{\text{physical interactions}}$ (%)	$CI_{\text{chemical cross-linking and physical interactions}} - CI_{\text{physical interactions}} = CI_{\text{chemical cross-linking (Shiff base)}}$ (%)
1	2	3	4	5
P1	0.25:1	42.27±0.1	16.47±2.5	25.8
P2	0.375:1	49.05±9.3	17.97±2.3	31.08
P3	0.5:1	61.83±7.3	23.89±3.7	37.94
